# 2-(4-Fluoro­phen­yl)-2-oxoethyl 4-meth­oxy­benzoate

**DOI:** 10.1107/S1600536811049233

**Published:** 2011-11-30

**Authors:** Hoong-Kun Fun, Wan-Sin Loh, B. Garudachari, Arun M. Isloor, M. N. Satyanarayan

**Affiliations:** a X-ray Crystallography Unit, School of Physics, Universiti Sains Malaysia, 11800 USM, Penang, Malaysia; b Organic Electronics Division, Department of Chemistry, National Institute of Technology-Karnataka, Surathkal, Mangalore 575 025, India; c Department of Physics, National Institute of Technology-Karnataka, Surathkal, Mangalore 575 025, India

## Abstract

In the title compound, C_16_H_13_FO_4_, the dihedral angle between the benzene rings is 84.28 (8)°. In the crystal, C—H⋯F and C—H⋯O hydrogen bonds link the mol­ecules to form a three-dimensional network. The crystal structure is consolidated by C—H⋯π inter­actions and short F⋯F contacts [2.7748 (14) Å] also occur.

## Related literature

For related structures and background to phenacyl benzoates, see: Fun *et al.* (2011**a* ▶,b* ▶). For the stability of the temperature controller used in the data collection, see: Cosier & Glazer (1986 ▶).
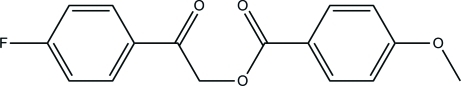


## Experimental

### 

#### Crystal data

C_16_H_13_FO_4_
*M* *_r_* = 288.26
Monoclinic, 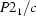

*a* = 9.3523 (2) Å
*b* = 10.1949 (2) Å
*c* = 15.6465 (4) Å
β = 118.842 (2)°
*V* = 1306.77 (5) Å^3^
*Z* = 4
Mo *K*α radiation
μ = 0.11 mm^−1^
*T* = 100 K
0.29 × 0.25 × 0.15 mm

#### Data collection

Bruker SMART APEXII CCD diffractometer
Absorption correction: multi-scan (*SADABS*; Bruker, 2009 ▶) *T*_min_ = 0.967, *T*_max_ = 0.984
18005 measured reflections
4669 independent reflections
3194 reflections with *I* > 2σ(*I*)
*R*_int_ = 0.061

#### Refinement

*R*[*F* ^2^ > 2σ(*F* ^2^)] = 0.055
*wR*(*F* ^2^) = 0.151
*S* = 1.03
4669 reflections
191 parameters
H-atom parameters constrained
Δρ_max_ = 0.53 e Å^−3^
Δρ_min_ = −0.29 e Å^−3^

### 

Data collection: *APEX2* (Bruker, 2009 ▶); cell refinement: *SAINT* (Bruker, 2009 ▶); data reduction: *SAINT*; program(s) used to solve structure: *SHELXTL* (Sheldrick, 2008 ▶); program(s) used to refine structure: *SHELXTL*; molecular graphics: *SHELXTL*; software used to prepare material for publication: *SHELXTL* and *PLATON* (Spek, 2009 ▶).

## Supplementary Material

Crystal structure: contains datablock(s) global, I. DOI: 10.1107/S1600536811049233/hb6504sup1.cif

Structure factors: contains datablock(s) I. DOI: 10.1107/S1600536811049233/hb6504Isup2.hkl

Supplementary material file. DOI: 10.1107/S1600536811049233/hb6504Isup3.cml

Additional supplementary materials:  crystallographic information; 3D view; checkCIF report

## Figures and Tables

**Table 1:** Hydrogen-bond geometry (Å, °) *Cg*1 and *Cg*2 are the centroids of the C1–C6 and C10–C15 rings, respectively.

*D*—H⋯*A*	*D*—H	H⋯*A*	*D*⋯*A*	*D*—H⋯*A*
C4—H4*A*⋯F1^i^	0.95	2.55	3.1334 (17)	120
C8—H8*B*⋯O3^ii^	0.99	2.43	3.3517 (19)	154
C11—H11*A*⋯O4^iii^	0.95	2.53	3.380 (2)	150
C1—H1*A*⋯*Cg*2^iv^	0.95	2.59	3.3693 (17)	139
C8—H8*A*⋯*Cg*1^v^	0.99	2.83	3.5309 (19)	128

Symmetry codes: (i) 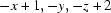; (ii) 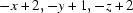; (iii) 
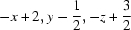
; (iv) 
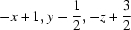
; (v) 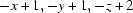.

**Fig. 1.**
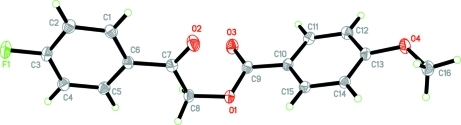
The molecular structure of the title compound, showing 50% probability displacement ellipsoids.

**Fig. 2.**
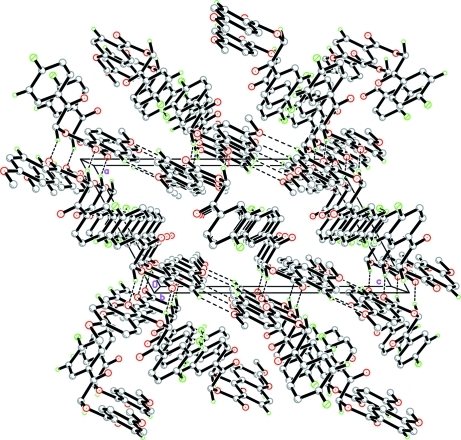
The crystal packing of the title compound, viewed along the b axis, showing the three-dimensional network. H atoms not involved in the intermolecular interactions (dashed lines) have been omitted for clarity.

## supplementary crystallographic information

### Comment

As part of our ongoing studies of phenacyl benzoates (Fun *et al.*, 2011*a*,*b*), we now report the synthesis and structure of the title compound.

In the title compound (Fig. 1), the dihedral angle formed between the fluoro-substituted (C1–C6) and the methoxy-substituted (C10–C15) benzene rings is 84.28 (8)°. Bond lengths and angles are within the normal ranges and are comparable to the related structures (Fun *et al.*, 2011**a*,b*).

In the crystal (Fig. 2), C4—H4A···F1, C8—H8B···O3 and C11—H11A···O4 hydrogen bonds (Table 1) link the molecules together to form a three-dimensional network. The crystal structure is further stabilized by C–H···π interactions (Table 1) involving the fluoro-substituted (*Cg*1) and the methoxy-substituted (*Cg*2) benzene rings.

### Experimental

A mixture of 4-methoxybenzoic acid (1.0 g, 0.0065 mol), potassium carbonate (0.99 g, 0.0072 mol) and 2-bromo-1-(4-fluorophenyl)ethanone (1.41 g, 0.0065 mol) in dimethylformamide (10 ml) was stirred at room temperature for 2 h. On cooling, colourless needle-shaped crystals of 2-(4-fluorophenyl)-2-oxoethyl 4-methoxybenzoate began to separate. It was collected by filtration and recrystallized from ethanol to yield yellow blocks of (I). Yield: 1.72 g, 91.0%. *M.p*: 387–388 K.

### Refinement

All H atoms were positioned geometrically and refined with a riding model with *U*_iso_(H) = 1.2 or 1.5 *U*_eq_(C) [C–H = 0.95 or 0.99 Å]. A rotating group model was applied to the methyl group.

### Crystal data

**Table:**

C_16_H_13_FO_4_	*F*(000) = 600
*M**_r_* = 288.26	*D*_x_ = 1.465 Mg m^−^^3^
Monoclinic, *P*2_1_/*c*	Mo *K*α radiation, λ = 0.71073 Å
Hall symbol: -P 2ybc	Cell parameters from 3370 reflections
*a* = 9.3523 (2) Å	θ = 2.5–32.1°
*b* = 10.1949 (2) Å	µ = 0.11 mm^−^^1^
*c* = 15.6465 (4) Å	*T* = 100 K
β = 118.842 (2)°	Block, yellow
*V* = 1306.77 (5) Å^3^	0.29 × 0.25 × 0.15 mm
*Z* = 4	

### Data collection

**Table:**

Bruker SMART APEXII CCD diffractometer	4669 independent reflections
Radiation source: fine-focus sealed tube	3194 reflections with *I* > 2σ(*I*)
graphite	*R*_int_ = 0.061
φ and ω scans	θ_max_ = 32.4°, θ_min_ = 2.5°
Absorption correction: multi-scan (*SADABS*; Bruker, 2009)	*h* = −14→14
*T*_min_ = 0.967, *T*_max_ = 0.984	*k* = −15→10
18005 measured reflections	*l* = −23→23

### Refinement

**Table:**

Refinement on *F*^2^	Primary atom site location: structure-invariant direct methods
Least-squares matrix: full	Secondary atom site location: difference Fourier map
*R*[*F*^2^ > 2σ(*F*^2^)] = 0.055	Hydrogen site location: inferred from neighbouring sites
*wR*(*F*^2^) = 0.151	H-atom parameters constrained
*S* = 1.03	*w* = 1/[σ^2^(*F*_o_^2^) + (0.0735*P*)^2^ + 0.1795*P*] where *P* = (*F*_o_^2^ + 2*F*_c_^2^)/3
4669 reflections	(Δ/σ)_max_ < 0.001
191 parameters	Δρ_max_ = 0.53 e Å^−^^3^
0 restraints	Δρ_min_ = −0.29 e Å^−^^3^

### Special details

**Table:**

Experimental. The crystal was placed in the cold stream of an Oxford Cryosystems Cobra open-flow nitrogen cryostat (Cosier & Glazer, 1986) operating at 100.0 (1) K.
Geometry. All e.s.d.'s (except the e.s.d. in the dihedral angle between two l.s. planes) are estimated using the full covariance matrix. The cell e.s.d.'s are taken into account individually in the estimation of e.s.d.'s in distances, angles and torsion angles; correlations between e.s.d.'s in cell parameters are only used when they are defined by crystal symmetry. An approximate (isotropic) treatment of cell e.s.d.'s is used for estimating e.s.d.'s involving l.s. planes.
Refinement. Refinement of *F*^2^ against ALL reflections. The weighted *R*-factor *wR* and goodness of fit *S* are based on *F*^2^, conventional *R*-factors *R* are based on *F*, with *F* set to zero for negative *F*^2^. The threshold expression of *F*^2^ > σ(*F*^2^) is used only for calculating *R*-factors(gt) *etc*. and is not relevant to the choice of reflections for refinement. *R*-factors based on *F*^2^ are statistically about twice as large as those based on *F*, and *R*- factors based on ALL data will be even larger.

### Fractional atomic coordinates and isotropic or equivalent isotropic displacement parameters (Å^2^)

**Table:**

	*x*	*y*	*z*	*U*_iso_*/*U*_eq_	
F1	0.36713 (11)	0.06839 (8)	0.92948 (7)	0.0261 (2)	
O1	0.82633 (13)	0.73381 (9)	0.99094 (8)	0.0196 (2)	
O2	0.58433 (13)	0.61282 (11)	0.84358 (8)	0.0266 (3)	
O3	0.94829 (13)	0.65098 (10)	0.90861 (8)	0.0242 (2)	
O4	0.85652 (13)	1.24512 (10)	0.77160 (8)	0.0223 (2)	
C1	0.44544 (17)	0.37022 (14)	0.84080 (11)	0.0197 (3)	
H1A	0.4037	0.4188	0.7818	0.024*	
C2	0.37612 (18)	0.25071 (14)	0.84149 (11)	0.0207 (3)	
H2A	0.2882	0.2159	0.7837	0.025*	
C3	0.43844 (18)	0.18361 (13)	0.92864 (11)	0.0192 (3)	
C4	0.56758 (18)	0.22904 (14)	1.01457 (11)	0.0203 (3)	
H4A	0.6071	0.1801	1.0733	0.024*	
C5	0.63790 (18)	0.34841 (14)	1.01258 (11)	0.0192 (3)	
H5A	0.7281	0.3810	1.0703	0.023*	
C6	0.57633 (16)	0.42079 (13)	0.92587 (10)	0.0170 (3)	
C7	0.64362 (17)	0.55037 (13)	0.91908 (10)	0.0176 (3)	
C8	0.79205 (17)	0.60283 (13)	1.00908 (11)	0.0187 (3)	
H8A	0.7709	0.6026	1.0653	0.022*	
H8B	0.8874	0.5458	1.0253	0.022*	
C9	0.89159 (16)	0.74423 (13)	0.93063 (10)	0.0177 (3)	
C10	0.88512 (16)	0.87944 (13)	0.89496 (10)	0.0162 (3)	
C11	0.96253 (17)	0.90621 (14)	0.83957 (11)	0.0186 (3)	
H11A	1.0230	0.8393	0.8290	0.022*	
C12	0.95157 (17)	1.02951 (14)	0.80006 (11)	0.0199 (3)	
H12A	1.0049	1.0474	0.7628	0.024*	
C13	0.86196 (16)	1.12769 (13)	0.81507 (10)	0.0173 (3)	
C14	0.78543 (17)	1.10306 (13)	0.87092 (10)	0.0170 (3)	
H14A	0.7259	1.1704	0.8818	0.020*	
C15	0.79728 (16)	0.97864 (13)	0.91055 (10)	0.0170 (3)	
H15A	0.7451	0.9611	0.9485	0.020*	
C16	0.7667 (2)	1.34901 (14)	0.78505 (12)	0.0240 (3)	
H16A	0.7724	1.4275	0.7507	0.036*	
H16B	0.8136	1.3683	0.8548	0.036*	
H16C	0.6525	1.3224	0.7589	0.036*	

### Atomic displacement parameters (Å^2^)

**Table:**

	*U*^11^	*U*^22^	*U*^33^	*U*^12^	*U*^13^	*U*^23^
F1	0.0259 (5)	0.0168 (4)	0.0324 (5)	−0.0055 (3)	0.0114 (4)	0.0027 (4)
O1	0.0248 (5)	0.0132 (4)	0.0226 (5)	−0.0019 (4)	0.0127 (4)	0.0008 (4)
O2	0.0247 (5)	0.0252 (5)	0.0243 (6)	−0.0029 (4)	0.0074 (4)	0.0073 (4)
O3	0.0250 (5)	0.0178 (5)	0.0330 (6)	0.0038 (4)	0.0166 (5)	0.0028 (4)
O4	0.0279 (6)	0.0165 (5)	0.0305 (6)	0.0026 (4)	0.0203 (5)	0.0046 (4)
C1	0.0189 (6)	0.0205 (6)	0.0190 (6)	0.0015 (5)	0.0087 (5)	0.0032 (5)
C2	0.0177 (6)	0.0206 (7)	0.0207 (7)	−0.0013 (5)	0.0068 (5)	−0.0009 (5)
C3	0.0193 (6)	0.0136 (6)	0.0261 (7)	−0.0010 (5)	0.0119 (6)	0.0000 (5)
C4	0.0227 (7)	0.0173 (6)	0.0196 (7)	0.0014 (5)	0.0091 (6)	0.0029 (5)
C5	0.0198 (6)	0.0170 (6)	0.0191 (6)	0.0002 (5)	0.0080 (5)	0.0001 (5)
C6	0.0164 (6)	0.0160 (6)	0.0203 (6)	−0.0001 (5)	0.0100 (5)	0.0006 (5)
C7	0.0167 (6)	0.0165 (6)	0.0204 (6)	0.0023 (5)	0.0096 (5)	0.0016 (5)
C8	0.0218 (6)	0.0134 (5)	0.0201 (7)	−0.0006 (5)	0.0093 (5)	0.0023 (5)
C9	0.0150 (6)	0.0164 (6)	0.0198 (6)	−0.0012 (5)	0.0069 (5)	−0.0003 (5)
C10	0.0141 (6)	0.0144 (6)	0.0181 (6)	−0.0007 (5)	0.0062 (5)	−0.0003 (5)
C11	0.0157 (6)	0.0173 (6)	0.0228 (7)	0.0002 (5)	0.0094 (5)	−0.0011 (5)
C12	0.0184 (6)	0.0205 (6)	0.0252 (7)	−0.0010 (5)	0.0140 (6)	0.0003 (6)
C13	0.0174 (6)	0.0150 (6)	0.0190 (6)	−0.0016 (5)	0.0084 (5)	0.0009 (5)
C14	0.0177 (6)	0.0150 (6)	0.0198 (6)	0.0004 (5)	0.0103 (5)	−0.0006 (5)
C15	0.0175 (6)	0.0167 (6)	0.0173 (6)	−0.0017 (5)	0.0087 (5)	−0.0008 (5)
C16	0.0302 (8)	0.0172 (6)	0.0301 (8)	0.0041 (6)	0.0188 (7)	0.0034 (6)

### Geometric parameters (Å, °)

**Table:**

F1—C3	1.3541 (16)	C7—C8	1.520 (2)
O1—C9	1.3528 (18)	C8—H8A	0.9900
O1—C8	1.4333 (16)	C8—H8B	0.9900
O2—C7	1.2150 (17)	C9—C10	1.4773 (19)
O3—C9	1.2165 (17)	C10—C15	1.3966 (19)
O4—C13	1.3657 (16)	C10—C11	1.3983 (19)
O4—C16	1.4290 (18)	C11—C12	1.383 (2)
C1—C2	1.383 (2)	C11—H11A	0.9500
C1—C6	1.401 (2)	C12—C13	1.396 (2)
C1—H1A	0.9500	C12—H12A	0.9500
C2—C3	1.378 (2)	C13—C14	1.3936 (19)
C2—H2A	0.9500	C14—C15	1.3926 (19)
C3—C4	1.384 (2)	C14—H14A	0.9500
C4—C5	1.391 (2)	C15—H15A	0.9500
C4—H4A	0.9500	C16—H16A	0.9800
C5—C6	1.401 (2)	C16—H16B	0.9800
C5—H5A	0.9500	C16—H16C	0.9800
C6—C7	1.4889 (19)		
			
C9—O1—C8	115.50 (11)	H8A—C8—H8B	108.2
C13—O4—C16	117.37 (11)	O3—C9—O1	122.76 (13)
C2—C1—C6	120.98 (13)	O3—C9—C10	124.49 (13)
C2—C1—H1A	119.5	O1—C9—C10	112.75 (12)
C6—C1—H1A	119.5	C15—C10—C11	119.40 (13)
C3—C2—C1	117.96 (14)	C15—C10—C9	122.01 (13)
C3—C2—H2A	121.0	C11—C10—C9	118.50 (12)
C1—C2—H2A	121.0	C12—C11—C10	120.38 (13)
F1—C3—C2	117.70 (13)	C12—C11—H11A	119.8
F1—C3—C4	118.95 (13)	C10—C11—H11A	119.8
C2—C3—C4	123.34 (13)	C11—C12—C13	119.84 (13)
C3—C4—C5	118.14 (13)	C11—C12—H12A	120.1
C3—C4—H4A	120.9	C13—C12—H12A	120.1
C5—C4—H4A	120.9	O4—C13—C14	124.28 (12)
C4—C5—C6	120.31 (13)	O4—C13—C12	115.20 (12)
C4—C5—H5A	119.8	C14—C13—C12	120.52 (13)
C6—C5—H5A	119.8	C15—C14—C13	119.25 (13)
C5—C6—C1	119.25 (13)	C15—C14—H14A	120.4
C5—C6—C7	123.09 (13)	C13—C14—H14A	120.4
C1—C6—C7	117.66 (12)	C14—C15—C10	120.60 (13)
O2—C7—C6	121.60 (13)	C14—C15—H15A	119.7
O2—C7—C8	120.06 (13)	C10—C15—H15A	119.7
C6—C7—C8	118.34 (12)	O4—C16—H16A	109.5
O1—C8—C7	109.63 (11)	O4—C16—H16B	109.5
O1—C8—H8A	109.7	H16A—C16—H16B	109.5
C7—C8—H8A	109.7	O4—C16—H16C	109.5
O1—C8—H8B	109.7	H16A—C16—H16C	109.5
C7—C8—H8B	109.7	H16B—C16—H16C	109.5
			
C6—C1—C2—C3	0.7 (2)	C8—O1—C9—C10	165.35 (11)
C1—C2—C3—F1	178.20 (13)	O3—C9—C10—C15	170.19 (14)
C1—C2—C3—C4	−0.9 (2)	O1—C9—C10—C15	−9.49 (19)
F1—C3—C4—C5	−179.17 (13)	O3—C9—C10—C11	−6.4 (2)
C2—C3—C4—C5	−0.1 (2)	O1—C9—C10—C11	173.95 (12)
C3—C4—C5—C6	1.2 (2)	C15—C10—C11—C12	−0.4 (2)
C4—C5—C6—C1	−1.3 (2)	C9—C10—C11—C12	176.28 (13)
C4—C5—C6—C7	178.76 (13)	C10—C11—C12—C13	−0.4 (2)
C2—C1—C6—C5	0.3 (2)	C16—O4—C13—C14	0.3 (2)
C2—C1—C6—C7	−179.77 (13)	C16—O4—C13—C12	−179.80 (13)
C5—C6—C7—O2	−177.62 (14)	C11—C12—C13—O4	−178.93 (13)
C1—C6—C7—O2	2.5 (2)	C11—C12—C13—C14	1.0 (2)
C5—C6—C7—C8	3.5 (2)	O4—C13—C14—C15	179.01 (13)
C1—C6—C7—C8	−176.36 (12)	C12—C13—C14—C15	−0.9 (2)
C9—O1—C8—C7	−73.21 (15)	C13—C14—C15—C10	0.2 (2)
O2—C7—C8—O1	7.51 (19)	C11—C10—C15—C14	0.4 (2)
C6—C7—C8—O1	−173.62 (11)	C9—C10—C15—C14	−176.08 (13)
C8—O1—C9—O3	−14.3 (2)		

### Hydrogen-bond geometry (Å, °)

**Table:**

Cg1 and Cg2 are the centroids of the C1–C6 and C10–C15 rings, respectively.

**Table:**

*D*—H···*A*	*D*—H	H···*A*	*D*···*A*	*D*—H···*A*
C4—H4A···F1^i^	0.95	2.55	3.1334 (17)	120
C8—H8B···O3^ii^	0.99	2.43	3.3517 (19)	154
C11—H11A···O4^iii^	0.95	2.53	3.380 (2)	150
C1—H1A···Cg2^iv^	0.95	2.59	3.3693 (17)	139
C8—H8A···Cg1^v^	0.99	2.83	3.5309 (19)	128

Symmetry codes: (i) −*x*+1, −*y*, −*z*+2; (ii) −*x*+2, −*y*+1, −*z*+2; (iii) −*x*+2, *y*−1/2, −*z*+3/2; (iv) −*x*+1, *y*−1/2, −*z*+3/2; (v) −*x*+1, −*y*+1, −*z*+2.
